# Surgical Orthodontic Treatment of Severe Skeletal Class II

**DOI:** 10.1155/2013/397809

**Published:** 2013-03-19

**Authors:** Fahad F. Alsulaimani, Maisa O. Al-Sebaei, Ahmed R. Afify

**Affiliations:** ^1^Orthodontic Division, Department of Preventive Dental Sciences, Faculty of Dentistry, King Abdul-Aziz University, Jeddah, Saudi Arabia; ^2^Oral and Maxillofacial Surgery Department, Faculty of Dentistry, King Abdul-Aziz University, Jeddah, Saudi Arabia

## Abstract

This paper describes an adult Saudi male patient who presented with a severe skeletal class II deformity. The case was managed with a combination of presurgical orthodontic treatment followed by a double jaw orthognathic surgery and then another phase of orthodontic treatment for final occlusal detailing. Extraction of the four first premolars was done during the presurgical orthodontic phase of treatment to decompensate upper and lower incisors and to give room for surgical setback of the maxillary anterior segment. Double jaw surgery was performed: bilateral sagittal split ramus osteotomy for 8 mm mandibular advancement combined with three-piece Le Fort I maxillary osteotomy, 6 mm setback of the anterior segment, 8 mm impaction of the maxilla, and 5 mm advancement genioplasty. Although the anteroposterior discrepancy and the facial convexity were so severe, highly acceptable results were obtained, both esthetically as well as occlusally.

## 1. Introduction

Orthognathic surgery is considered for the treatment of dentofacial skeletal deformities for more than 100 years ago. Interestingly, the first jaw deformity correction was performed without anesthesia in the United States by Simon Hullihen, an American general surgeon, in the mid of the 19th century.

Dentofacial skeletal deformities always cause severe functional and esthetic problems to the patient. In adult severe cases, the combined approach, orthodontic and orthognathic surgery, is always the treatment of choice, and the results obtained usually ensure a better esthetic, functional, and stable results [1–5].

Class II skeletal deformity is characterized by an exaggerated sagittal distance between the maxilla and the mandible, which could be the result of maxillary prognathism, mandibular retrognathism, or both.

Presurgical orthodontic decompensation is essential to enable the surgeon to make a considerable amount of surgical correction, otherwise the esthetic and functional outcome of the entire procedure will not be that ideal [1–3].

## 2. Case Report

A 21-year-old Saudi male was referred to the orthodontic clinic, Faculty of Dentistry, King Abdul-Aziz University for the treatment of “Bothering anterior teeth.” At the first consultation visit, the patient expressed his great concerns about his anterior teeth in addition to his severely retruded chin. His medical history was nonrelevant except for a scar resulting from closure of an upper-left unilateral cleft lip.

**Figure 1:**
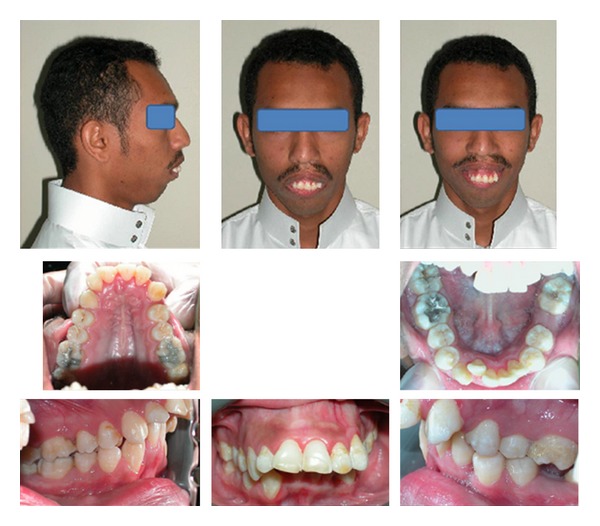
Pretreatment extraoral and intraoral photographs.

The clinical examination of the patient revealed a severe skeletal class II pattern with a severe mandibular retrognathism. The frontal facial view showed a mesofacial pattern, slightly deviated nose to the right, an excessive lower face height, and an interlabial gap of 21 mm. The interpupillary line was parallel to the occlusal plan, and the lips were incompetent at rest with the lower lip resting behind the upper incisors. At rest, there is a 10 mm incisal show in addition to 4 mm of the gum. The lips are incompetent at rest with a short upper lip, while the lower lip is resting behind the upper incisors. The upper midline is deviated to the right by 3 mm. Upon smiling, there was a severe gingival show around 12 mm. The lateral view of the face revealed an average nose, a normal nasolabial angle, a convex profile, a severe mandibular retrognathism, a severely deficient chin (Figure 1).

**Figure 2:**
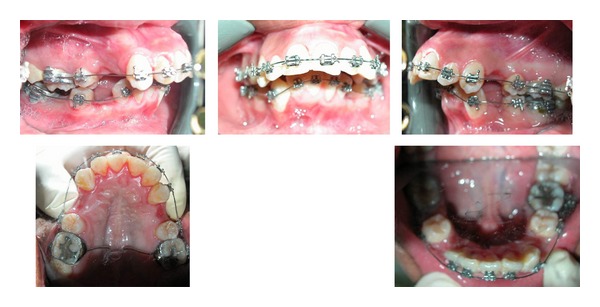
Presurgical intraoral photographs.

Intraoral photographs reveal a class II molar and canine relation on the right side, while on the left side it is undetermined due to the missing lower left first permanent molar. The upper arch is V shaped, while the lower arch is U shaped. There is around 5 mm lower anterior crowding, meanwhile there is around 6 mm anterior spacing in the upper arch. There is an excessive overjet, almost 15 mm. There is an exaggerated lower curve of Spee causing an impinging overbite with markedly distinct occlusal planes in the anterior and posterior segments (Figure 2).

Radiographically, the panoramic view revealed a normal bony trabeculation, the full number of permanent teeth except for an extracted lower-left first molar, and impacted upper and lower third molars. Cephalometric analysis revealed that the patient had a severe skeletal class II, and the ANB angle was 16° which is more resorted to the lower jaw. Upper incisor position was proclined and protruded, while the lower incisors were more severely proclined and protruded. Vertically, the patient had an increased lower face height. The chin was markedly deficient (Table 1).

After a complete diagnosis of the case, the patient was informed by the detailed treatment plan, and it was explained to the patient that the presurgical orthodontic preparation “decompensation” of the dentition will worsen the deformity and that the malocclusion, facial profile, and speech will be temporarily worsened. The patient was further informed that this presurgical treatment only improves the bony support for the teeth, and all the facial and profile changes will result after the upcoming surgical procedures.

## 3. Diagnosis

### 3.1. Treatment Objectives

The treatment objectives were to improve the patient's facial esthetics: patient's facial profile, mandibular retrognathism, increased lower third of the face, gummy smile, incompetent lips, dental midline shift, and normalizing the overbite and overjet.

### 3.2. Treatment Plan

Presurgical orthodontic phase aimed to decompensate upper and lower incisors via extraction of the four first premolars. Anchorage was maximized in the upper arch through the use of transpalatal arch (TPA) in addition to including the upper second molars.

### 3.3. Treatment Progress

#### 3.3.1. Presurgical Orthodontic Treatment

In general, the goal of presurgical orthodontics is to position the teeth, allowing an optimal surgical correction of the jaw bones. This will make the malocclusion look worse presurgically, but it will show the true entity of the skeletal problem, thus facilitating an optimal surgery [4]. Our aims from the presurgical treatment were to decompensate the upper and lower incisors and to level and align both arches and relief of crowding in the lower arch. Upper and lower first premolars were extracted to get space for retracting the lower incisors, alleviation of lower arch crowding, uprighting the upper incisors, and severing dental class II relation. The patient received 0.018-inch Roth edgewise appliance. Initial leveling was accomplished with 0.016-inch nickel-titanium (Ni-Ti) arch wires. Anchorage was maximized in the maxillary arch by inserting a TPA in addition to bonding the 2nd molars. After initial leveling and alignment, the upper and lower cuspids were retracted; lower incisors were decompensated, and a space left in the upper premolars area for anterior maxillary was set back.

#### 3.3.2. Orthognathic Surgery

*Preoperative Surgical Planning*. Upper and lower impressions were taken, and study casts were prepared. The models were mounted on a semi-adjustable articulator using a face-bow transfer. A full orthognathic model surgery was performed. Final and intermediate splints were fabricated using orthodontic cold-cure resin.

*Surgery. *A standard bilateral sagittal split incision was performed, and the medial aspect of the mandible was exposed. After identifying and protecting the inferior alveolar neurovascular bundle, a bilateral sagittal split osteotomy was performed with a surgical saw. 

A standard Le Fort I incision was performed in the mucosa. This was followed by a Le Fort I osteotomy using a surgical saw. The maxilla was downfractured and mobilized. A 3-piece maxillary osteotomy was performed using the surgical saw followed by a thin osteotome by cutting bilaterally in the mesial and distal of the extraction socket of the upper first bicuspid. A strip of bone measuring 6 mm was removed from each side. This was done to facilitate the posterior repositioning of the anterior segment of the maxilla. 

The maxillary segments were aligned and positioned in the intermediate splint. The patient's occlusion was placed into the intermediate splint, and the patient was placed in an intermaxillary fixation with wires and elastics. The maxilla was fixated in the new position using mini titanium alloy plates and 2.0 screws in the areas of the pyriform rim and maxillary buttress. The intermaxillary fixation and intermediate splint were removed.

Attention was then drawn back to the mandible where the bilateral sagittal split osteotomy was completed, the mandible was advanced to 8 mm, and the patient was placed in proper occlusion using the final splint. The mandible was fixated using mini titanium plates and 2.0 screws. The intermaxillary fixation was removed, and the occlusion was checked to be as predetermined in the model surgery preoperatively. The occlusion was passive and reproducible. An anterior 5 mm advancement genioplasty was performed. The intermaxillary fixation was placed again using elastics.

*Postoperative Care*. The wound was checked daily for one week for signs of ischemia. The splint was kept in place for 4 weeks, and the patient was placed on a liquid and pureed diet and sinus precautions. The splint was removed in the clinic, and the occlusion was checked. It was stable and reproducible.

#### 3.3.3. Postsurgical Orthodontic Treatment

**Figure 3:**
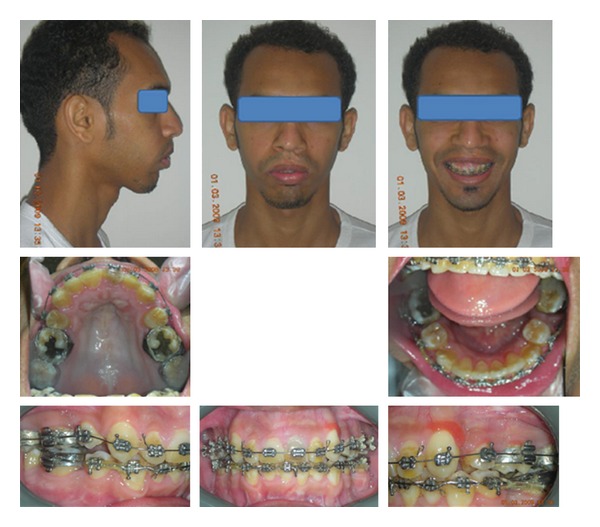
Postsurgical extraoral and intraoral photographs.

Postsurgical orthodontics was continued after surgery to close minor spaces distal to the cuspids in the upper and lower arches. The goals of this phase of treatment were to rehabilitate and restore the neuromuscular function and get final occlusal settling. Occlusal function and settling was greatly improved through the use of intermaxillary elastics. Occlusal selective grinding was also done to finalize the occlusion. The postsurgical phase of orthodontic treatment continued for 8 months (see Figure 3).

## 4. Results

Both the gummy smile and lips incompetence were greatly improved. The patient profile showed a marked improvement. 

**Figure 4:**
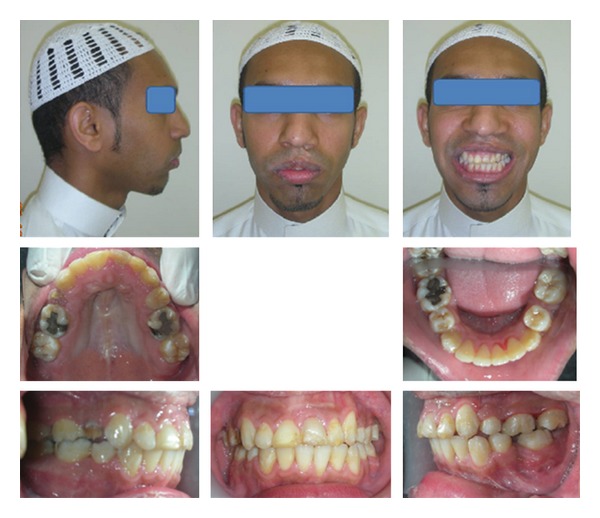
Posttreatment extraoral and intraoral photographs.

Although the occlusion and facial esthetics were greatly improved, the results were not that perfect. The patient started to develop some carious lesions. In addition to this, the patient started to feel distressed due to the lengthy treatment time. That is why we decided to debond, although the results were less than ideal, to enhance both the dental brushing as well as the fast psychological adaptation (see Figure 4). 

## 5. Discussion

The surgical correction of such severe dentofacial deformities is a functional and esthetic surgery that affects patients' self-perception. The patient appreciated the improvement in his facial appearance after orthognathic surgery that was associated with a noted improvement in his psychosocial adjustments.

**Figure 5:**
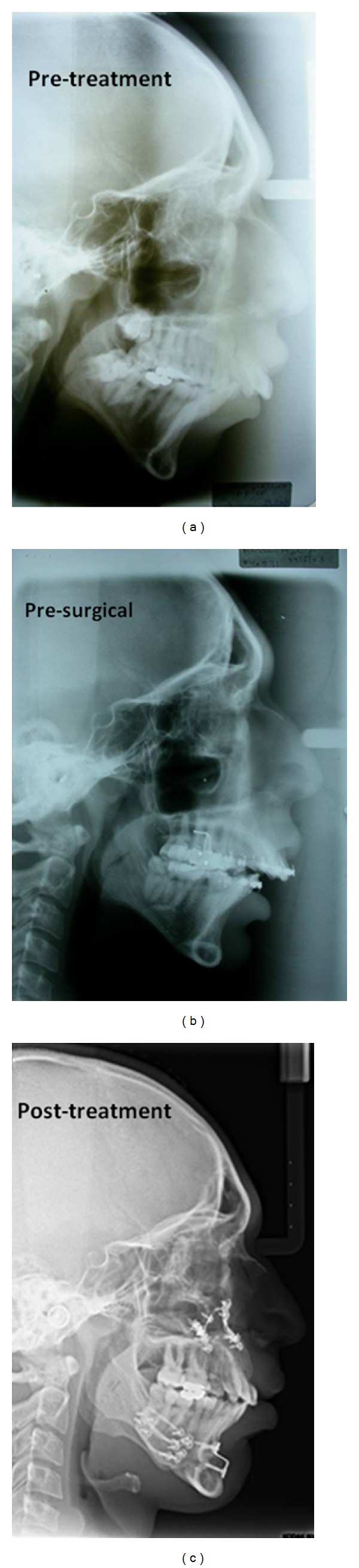
Pretreatment, presurgical and posttreatment lateral cephalograms.

Although there is new trends in the management of skeletal dentofacial deformities to start surgery then proceed the postsurgical phase of orthodontic treatment, we still believe in the presurgical orthodontic preparation as it adapts the occlusion greatly to the new postsurgical position and consequently a better opportunity for a more stable occlusion. Those who believe in starting with surgery are concerned about the severity of deterioration of both the facial profile and function during the period of presurgical orthodontics (see Figure 5).

Orthognathic surgery is only one part of the process to correct a dentofacial deformity. The process starts with the initial diagnosis, followed by a treatment plan, and then patient consent. Treatment generally begins with a dental assessment to correct decay, followed by orthodontic decompensation in preparation for surgical intervention. Orthognathic surgery is followed by postoperative orthodontia to maximize the occlusal relationship.

## Figures and Tables

**Table 1:** Cephalometric analysis.

Area of study	Measurement	Mean	Pretreatment	Posttreatment
Sagittal relationship	SNA	82°	87°	84°
	SNB	80°	71°	75°
	ANB	2°	16°	9°
	N Pg/FH	87°	73°	86°
	Wits appraisal	−1–0 mm	26 mm	5 mm
	Pg to NB dist.	2-3 mm	0 mm	3 mm

Vertical relationship (divergency)	Mand. Pl. to FH	25°	39°	33°
	Mand. Pl. to SN	32°	48°	45°
	*Y* axis S Gn/SN	60°–66°	77°	63°
	Lower face height	64 mm	85 mm	74 mm

Dental relationship (incisor position)	U Inc. to SN	103°	119°	109°
	U Inc. to NA	22°	32°	25°
	U Inc. to NA dist.	4 mm	6 mm	3 mm
	U Inc. to L Inc.	130°–132°	86°	109°
	L Inc. to Mand.	90°	105°	95°
	L Inc. to NB	25°	44°	36°
	L Inc. to NB dist.	4 mm	10 mm	10 mm

Soft tissue relationship	Upper lip to E-line	−4 mm	9.3 mm	3.8 mm
	Lower lip to E-line	−2 mm	7 mm	0 mm
	Nasolabial angle	90°–110°	77°	93°
